# Artificial intelligence in plant science: from image-based phenotyping to yield and trait prediction

**DOI:** 10.3389/fpls.2025.1732979

**Published:** 2026-01-29

**Authors:** Tong Wang, Ran Tong, Ting Xu, Yue Li, Yonghao Chen

**Affiliations:** 1 Department of Plant Science and Landscape Architecture, University of Connecticut, Storrs, CT, United States; 2 Mathematics and Statistics Department, University of Texas at Dallas, Richardson, TX, United States; 3 Department of Computer Science, University of Massachusetts Boston, Boston, MA, United States; 4 Department of Computer Science, Purdue University, West Lafayette, IN, United States; 5 San Diego Health, University of San Diego, San Diego, CA, United States

**Keywords:** AI, image analysis, phenotyping, precision agriculture, remote sensing, yield prediction

## Abstract

With the development of artificial intelligence (AI) in complicated imaging and remote sensing technologies, plant research is transitioning from manual measurements to automated data collecting. High-throughput image-based phenotyping enables the precise and automated acquisition of traits across various spatial and temporal scales, ranging from controlled laboratory settings to intricate field. Furthermore, AI facilitates the combination of satellite observations, unmanned aerial vehicle (UAV) imaging, soil and climate data, and spatiotemporal information to enhance the precision of trait monitoring and yield prediction. These advances enhance the ability to evaluate and predict crop performance under variable environmental conditions. This paper offers a cross-disciplinary paradigm for accurate and sustainable modern agriculture by merging AI methodologies with plant phenotyping and yield forecasting.

## 1 Introduction

Global food systems are increasingly challenged by rapid population growth, climate variability, and limited natural resources, creating an urgent need for innovative strategies to enhance crop productivity and ensure sustainable agricultural production (van Dijk et al., 2021). Conventional methods for phenotyping and yield forecasting are laborious and unable to capture complex interactions.

Artificial intelligence (AI) is now widely applied across scientific disciplines (Jumper et al., 2021; Pyzer-Knapp et al., 2022; Rajpurkar et al., 2022; Advancing geoscience with AI, 2024; Murphy et al., 2024; Serrano et al., 2024; Bodnar et al., 2025). In recent years, the utilization of machine learning (ML) and deep learning (DL) in agriculture has expanded swiftly, propelled by substantial methodological advancements in algorithm creation and the rising accessibility of extensive crop datasets (Joshi et al., 2023; Waqas et al., 2025). These approaches remedy long-standing weaknesses in lab and field by taking over tasks that are tedious or require expert handling (Fahlgren et al., 2015). Plant scientists are beginning to use more computer-driven procedures to automatically quantify image-based traits and predict crop yield based on data collected across the different locations. Since global farming is impacted by increasing food demands and shifting weather patterns, this change occurs at a pivotal time in world agriculture. Secure and high productivity of crops amid the environmental changes will demand agile and high yielding agricultural systems, which, are very difficult to achieve using traditional phenotyping and breeding strategies. The use of computational techniques minimizes time intervals between crops and allows for improved accuracy and lower down the repeat measurement of trait assessments (van Klompenburg et al., 2020; Crossa et al., 2025a).

**Figure 1:**
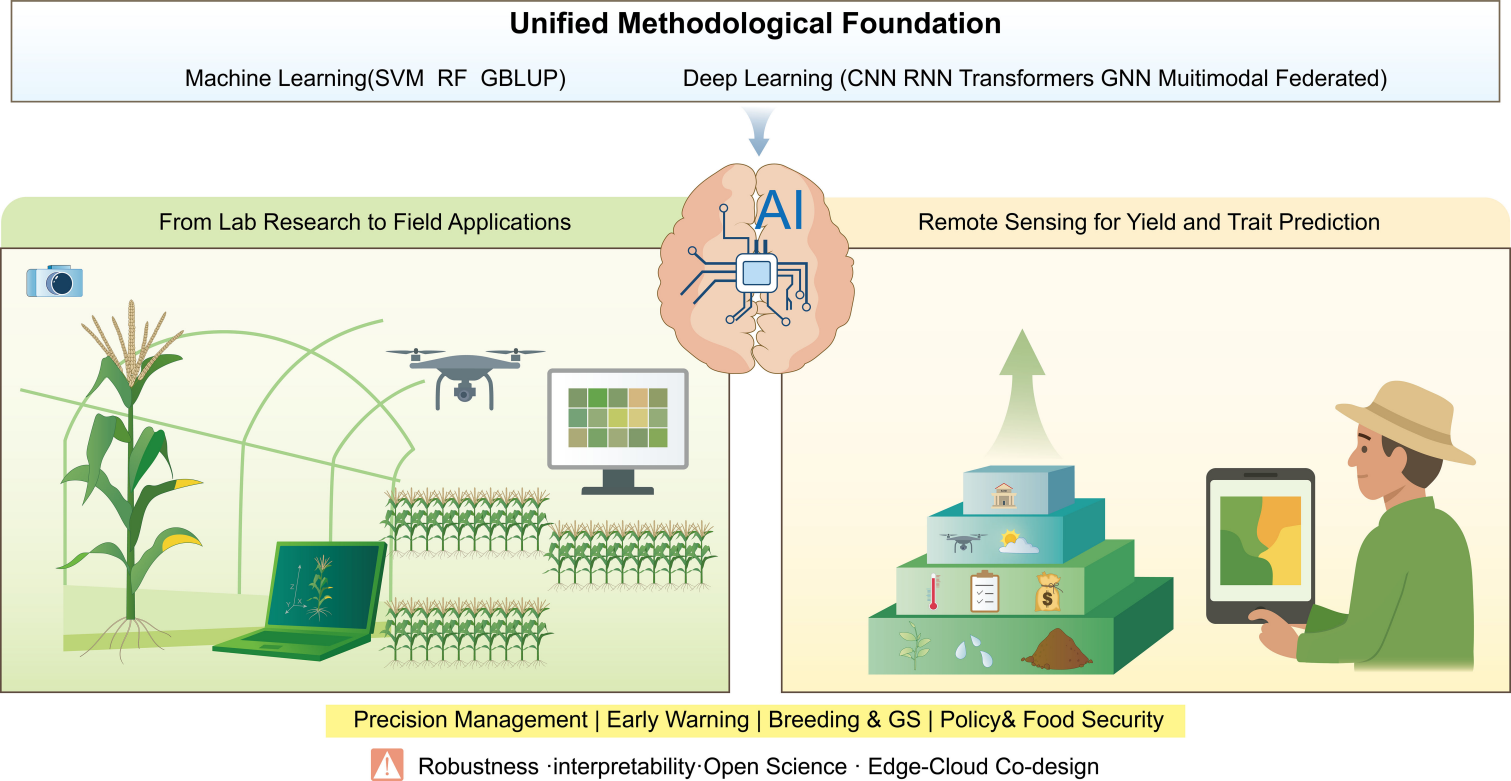
Conceptual framework of AI-enabled plant phenotyping and yield forecasting. The core structure and conceptual framework of the study. (1) Image-based phenotyping (2) Multi-Source remote sensing.

In this review we present a two-part summary on how AI has started to revolutionize plant science in: (i) Image-Based Phenotyping, where algorithms analyze plant images at varying scales from single organs to field canopies. Another application includes (ii) Yield and Trait Prediction through remote sensing, where a combination of varied environment factors like rainfall and temperature data is used to estimate crop yield. Increased adoption and usage of plant imaging provide a switch across scientific disciplines. With the ability of applying reliable and phenotypic information and integrating smart modeling tools, plant biologists can facilitate and improve development of crops; with the integration of high-throughput phenotypic data and advanced predictive algorithms with complicated environment data, the crop improvement cycle can be both fast tracked and enhanced in precision and efficacy (**Figure 1**).

The core structure and conceptual framework of the study (van Dijk et al., 2021). Image-based phenotyping (Rajpurkar et al., 2022) Multi-Source remote sensing.

## 2 Unified AI framework for plant phenotyping and trait analysis

**Figure 2:**
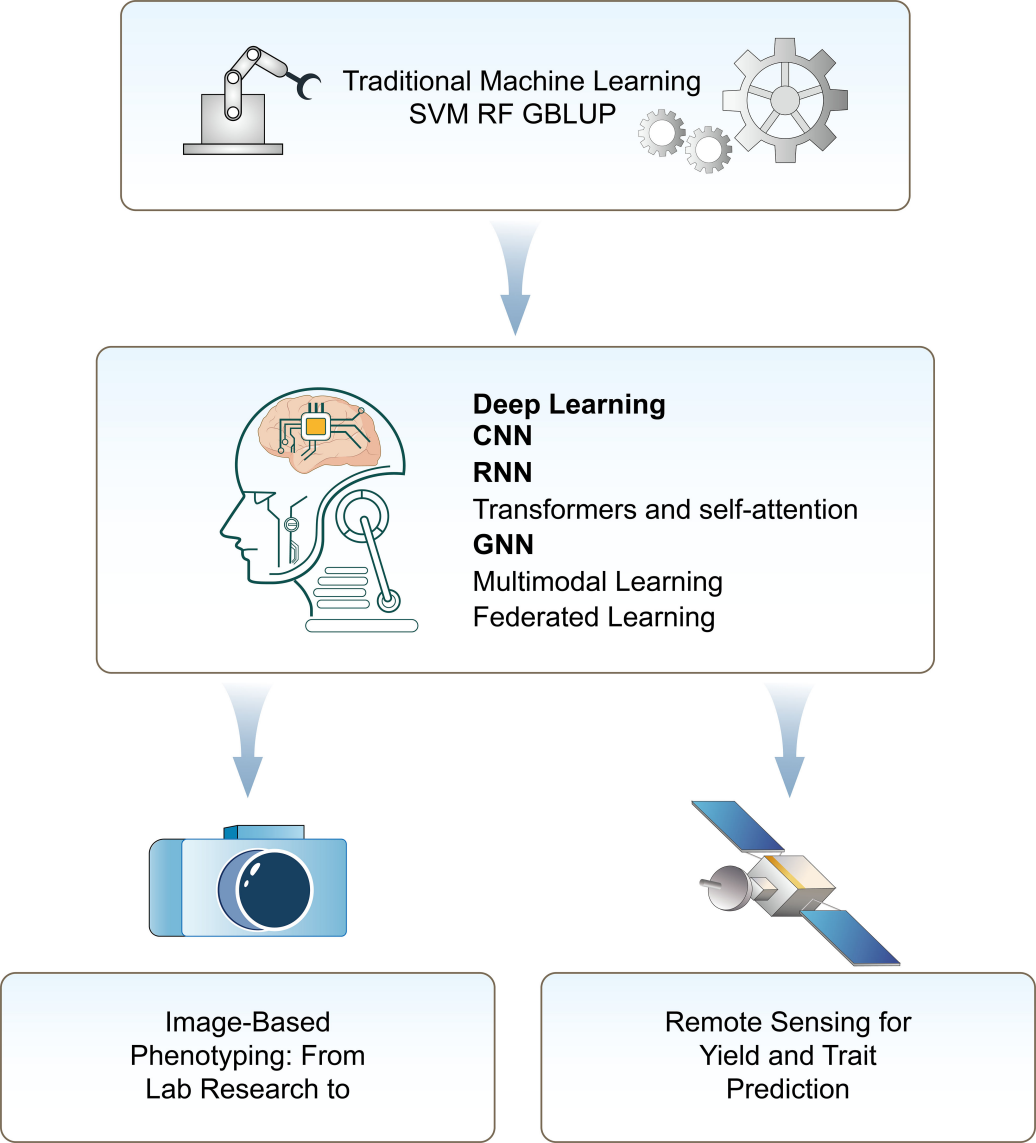
Framework of AI modeling in plant phenotyping and remote sensing. Traditional ML methods, such as SVM, RF, and GBLUP, for early phenotypic prediction tasks. With the advent of DL, advanced models including CNN, RNN, Transformer, GNN, multimodal learning, and FL for efficient and scalable data analysis for phenotyping and sensing.

Modern AI has moved from engineered features to end-to-end DL with automatic representation learning (**Figure 2**).

### 2.1 Traditional machine learning

Random forest (RF) is suitable for nonlinear, high-dimensional data and used well in trait prediction and remote sensing (Belgiu and Drăguţ, 2016), Support Vector Machine (SVM) improve generalization by maximizing class margins (Cortes and Vapnik, 1995), and bagging strengthens the robustness of RF (Breiman, 2001). Genomic Best Linear Unbiased Prediction (GBLUP) is a linear mixed model in genomic selection (Clark and van der Werf, 2013).

### 2.2 Deep feature learning

#### 2.2.1 Convolutional neural networks

CNNs, designed to capture 2D structural variation, perform well in handwritten character recognition. Graph transformer networks (GTNs) enable joint training of multiple modules and enhance performance. Early work such as LeNet illustrated the effectiveness of CNNs in learning hierarchical features from raw images (Lecun et al., 1998). Building on this foundation, AlexNet marked a major advance in large-scale image classification by leveraging graphics processing units (GPUs) and the rectified linear unit (ReLU) activation function (Krizhevsky et al., 2017). Subsequent CNN architectures further pushed performance: VGG deepened networks using small convolutional filters (Simonyan and Zisserman, 2015), and ResNet introduced residual connections that enabled very deep models (He et al., 2016). U-Net and related encoder-decoder models are effective for dense prediction tasks by combining multi-scale features (Ronneberger et al., 2015).

#### 2.2.2 Recurrent neural networks and sequence modeling

Recurrent Neural Networks (RNNs) incorporate feedback connections that create an internal memory of sequential data. Basic RNNs suffer from vanishing or exploding gradients when learning very long-term dependencies. Two gating architectures were introduced to mitigate this: the Long Short-Term Memory (LSTM) (Hochreiter and Schmidhuber, 1997). and the Gated Recurrent Unit (GRU) (Cho et al., 2014). Another pivotal innovation was the Attention mechanism, first applied to neural machine translation by Jointly Learning to Align and Translate (Bahdanau et al., 2014).

#### 2.2.3 Transformers and self-attention

The limitations of RNNs on long sequences led to the emergence of Transformers, which rely on self-attention to process entire sequences in parallel by relating each element to every other (Vaswani et al., 2017). BERT use bidirectional encoders to generate rich representations for downstream tasks (Devlin et al., 2019). In computer vision, Vision Transformers (ViT) demonstrated that one can treat an image as a sequence of patches and achieve competitive results using Transformer architectures (Dosovitskiy et al., 2021). Hierarchical variants such as the Video Shifted Window (Swin) Transformer introduce localized attention windows and multiscale processing to improve efficiency on high-resolution images (Liu et al., 2021).

#### 2.2.4 Graph neural networks

Graph Neural Networks (GNNs) extend deep learning to graph-structured data. One influential GNN variant is the Graph Convolutional Network (GCN) (Kipf and Welling, 2017), which generalizes the notion of convolution to graph neighborhoods. More broadly, Message Passing Neural Networks (MPNNs) provide a framework where messages sent along edges can be learned functions of both neighbor and edge features (Gilmer et al., 2017). The Graph Attention Network (GAT) introduces attention weights on edges (Velickovic et al., [[NoYear]]).

#### 2.2.5 Multimodal learning

Baltrusaitis et al. provides a comprehensive survey of multimodal Learning, tracing the progression from such independent processing to modern approaches that learn joint representations (Baltrusaitis et al., 2019). Multimodal deep learning integrates heterogeneous data types within a single framework and uses fusion and alignment strategies to learn shared representations, improving performance across diverse tasks (Shaban and Yousefi, 2024). Vision-and-language models like ViLBERT (Lu et al., 2019) and LXMERT (Tan and Bansal, 2019) are examples of vision-and-language models that have co-attentional transformer layers. These layers facilitate interaction and mutual influence between the picture areas and the language tokens. CLIP model exemplifies this methodology by training on image-text pairs to provide analogous embeddings for corresponding pairs and unique embeddings for non-corresponding pairs, allowing zero-shot transfer by enabling the model to learn a universal correspondence between images and natural language descriptions (Radford et al., 2021).

#### 2.2.6 Federated learning

Modern AI systems are being used in situations such as user smartphones or different universities and institutions, and can’t be combined directly because of privacy concerns. FL solves this problem by letting models be trained together without putting all the raw data in one place (McMahan et al., 2017). Federated Averaging (FedAvg) averages the model parameters from each client, making communication in federated networks more efficient (Kairouz et al., 2021).

Traditional ML methods, such as SVM, RF, and GBLUP, for early phenotypic prediction tasks. With the advent of DL, advanced models including CNN, RNN, Transformer, GNN, multimodal learning, and FL for efficient and scalable data analysis for phenotyping and sensing.

### 2.3 Methodological foundation of AI in plant phenotyping and remote sensing

There are trade-offs between convergence and accuracy (**Table 1**; **Figure 2**). This trend show that model design is moving in a more general, flexible, and real-world-aligned direction.

**Table 1:** Comparative summary of AI methods for plant phenotyping applications.

Method category	Representative model	Core principle	Advantages	Limitations	Representative literature
Machine Learning	SVM, RF, GBLUP	Tree-Based Ensemble Models and Linear Mixed Models	Good interpretability and strong robustness	Difficult to capture complex nonlinearity	Cortes and Vapnik, 1995; Breiman, 2001
Deep Learning	CNN	Local Feature Extraction	High accuracy and easy deployment	Insufficient global modeling capability	Krizhevsky, A. et al. 2012
Deep Learning	RNN	Temporal Dependency Modeling	Suitable for time-series data	High training cost and poor parallelizability	Cho et al., 2014
Deep Learning	Transformers and Self-Attention	Global Dependency and Efficient Representation Learning	Strong global modeling capability	High computational cost and memory footprint	Vaswani et al., 2017
Deep Learning	GNN	Graph-Structured Relationship Modeling	Suitable for complex relationship modeling	Performance sensitive to graph construction; limited scalability on large, dense graphs	Kipf and Welling, 2017; Velickovic et al., 2017
Deep Learning	Multimodal Learning	Multi-source Feature Joint/Joint Embedding	Strong cross-modal representation learning and generalization	Modality alignment and missing-modality handling remain challenging	Ngiam et al., 2011; Lu et al., 2019 (ViLBERT) Radford et al., 2021 (CLIP) Cem Akkus et al., 2023
Deep Learning	FL	Distributed Collaborative Learning with Data Locality	Non-IID data, communication overhead, and possible convergence degradation	Slow convergence and Accuracy Loss	McMahan et al., 2017; Kairouz et al., 2021

## 3 Image-based phenotyping: from lab research to field applications

Traditional plant phenotyping, which relies on manual measurements of plant height and leaf size, is slow, small in scale, and subjective. Modern image-based approaches enable nondestructive, large-scale trait acquisition through automated analysis. An early breakthrough in image-based plant phenotyping was achieved through automated segmentation of time-series plant images under variable backgrounds (Minervini et al., 2014). Advances in imaging hardware (high-resolution cameras, multi/hyperspectral sensors, depth sensors) and robotics now allow plants to be imaged from many angles and modalities, from controlled greenhouse to over field. The improvement of computer image analysis has made it much more accurate, allowing for the consistent extraction of morphological and physiological information across a wide range of temporal and spatial scale (Tardieu et al., 2017).

Modern image-based phenomics allows for the large-scale, non-invasive assessment of essential plant characteristics. By reviewing more than 200 studies on crop diseases, animal health, and aquaculture, researchers identified several common challenges in data and practical implementation. They emphasize the need for flexible, efficient, and scalable AI models to address these issues (Nawaz et al., 2025). Recent advancements in computer techniques have considerably improved the accuracy, efficiency, and scope of trait analysis. This change helps solve problems that have been around for a long time, such as manual scoring, changes in the environment, and low temporal resolution. More accurate trait characterization makes it easier to combine phenotypic data, which speeds up the identification of new traits and the development of crops (Tardieu et al., 2017; Jiang and Li, 2020; Nabwire et al., 2021).

**Figure 3:**
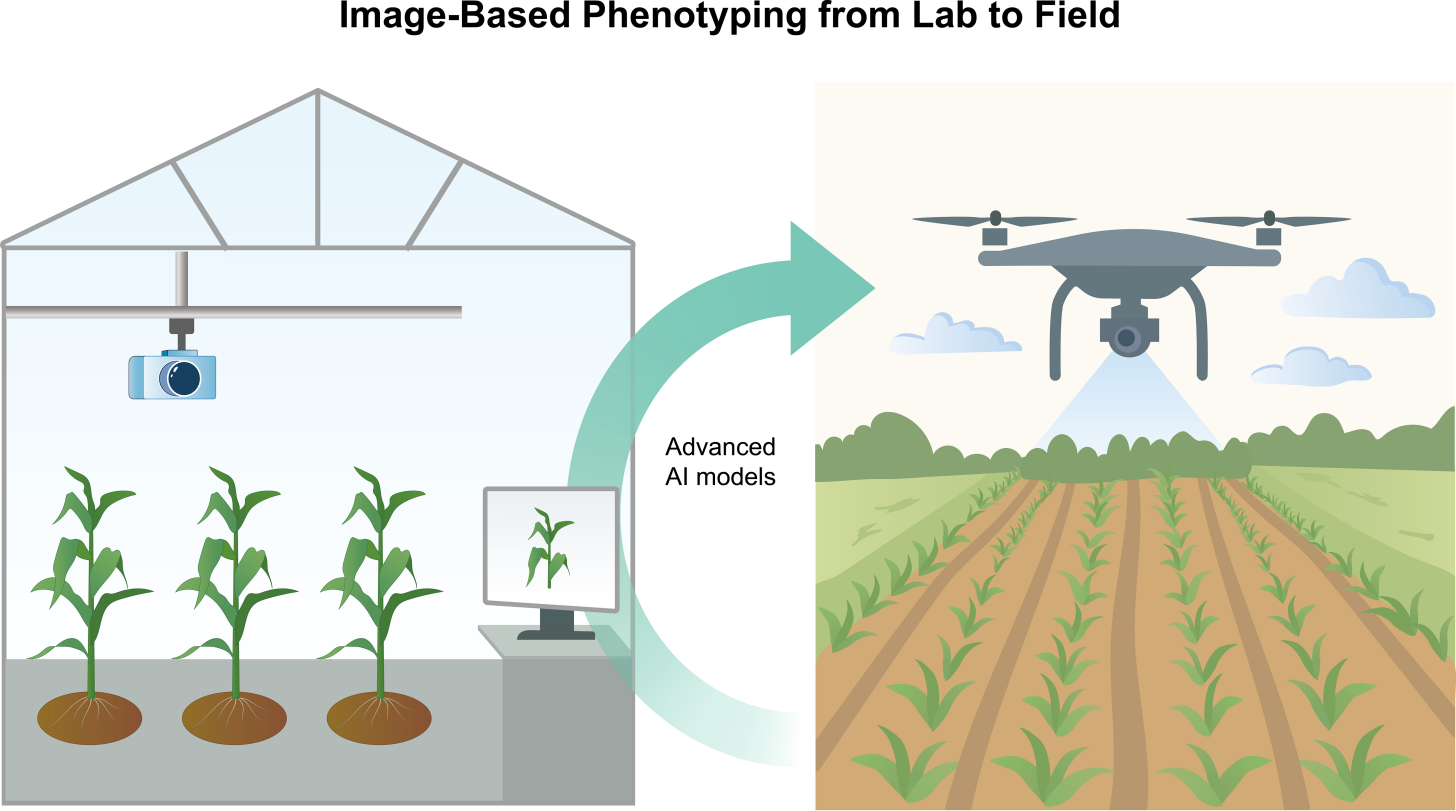
Overview of image-based phenotyping from lab to field. The controlled-environment phenotyping (left, e.g. greenhouse imaging systems with cameras on rails or conveyor belts) to field phenotyping (right, e.g. UAV drone surveys over crop plots).

This section reviews how imaging technologies and AI algorithms for plant phenotyping have progressed, and how they are being deployed from controlled lab settings to real-world field applications (**Figure 3**, **Table 2**).

**Table 2:** Representative AI-based image phenotyping applications.

Crop	Region	AI model	Data type	Performance metrics	Key findings	Reference
Multiple crops	Lab (global images)	CNN (AlexNet/GoogLeNet trained on PlantVillage)	RGB images of leaves	99.35% overall accuracy, F1 = 0.9934	99% accuracy in identifying 38 crop-disease classes from images, establishing a new automated plant disease diagnosis.	(Mohanty et al., 2016)
Multiple species (leaves)	Lab (leaf images database)	CNN (LeafNet)	RGB images of isolated leaves	86.3%, 95.8%, and 97.9% Top-1 accuracy on the LeafSnap, Foliage, and Flavia datasets, respectively.	Automated plant identification based on scans and smartphone pictures to learn leaf shape/texture features.	(Barré et al., 2017)
Arabidopsis	Lab (controlled environment)	CNN	Ara2012 and Ara2013-CanonRGB images	R2 = 0.42 (improved count accuracy *vs* prior methods)	Enhancing leaf counting accuracy in Arabidopsis seedlings and enabling reliable high-throughput phenotyping of leaf number.	Ubbens et al., 2018 (Ubbens and Stavness, 2017)
Clover	Greenhouse (controlled environment)	Faster R-CNN + U-Net (Greenotyper pipeline)	Time-lapse RGB images	97.98% bounding-box AP; 0.95pixel accuracy (IoU = 0.84)	Demonstrated a scalable, affordable high-throughput phenotyping system for plant growth traits.	(Tausen et al., 2020)
Rice (paddy weeds)	Hunan, China (rice fields)	MobileNetV2-UNet & FFB-BiSeNetV2 (lightweight CNN semantic segmentation)	UAV low-altitude RGB imagery	PA = 93.09%; weed IoU = 80.28%; mIoU = 64.38%	Real-time weed detection model via UAV in rice paddies, enabling timely weed mapping.	Lan et al., 2021
Fruits	Cross-dataset (orchards, greenhouse)	CNN-based detector + CNN-based GAN (CycleGAN + Improved-YOLOv3)	RGB images of fruit on trees (unlabeled target domain)	mAP = 87.5% (apple), 76.9% (tomato) after adaptation	Trained on orange images generalized well to apples and tomatoes, greatly bridging the species gap in fruit detection.	(Zhang et al., 2021)
Wheat	Outdoor field	DeepLabV3+ (CNN semantic segmentation)	UAV and ground RGB images (various wheat genotypes)	IoU of 0.77 and 0.90 for plants and soil, respectively (robust segmentation across genotypes)	Achieved robust pixel level plant-soil segmentation in field-grown wheat canopies across years and genotypes.	(Zenkl et al., 2021)
Various crops	Controlled environments	PSegNet (3D CNN–based point cloud segmentation network)	3D point clouds of plants (lidar scans)	mIoU = 89.9% mRec 90.15%, mCov 83.38%, mWCov 89.54%	For simultaneous segmentation of plant point clouds, improving the precision of 3D organ-level trait extraction.	Li et al., 2022
Multiple crops (diseases)	Field conditions	MobileViT (PMVT – lightweight Vision Transformer)	Field RGB images of plant leaves	Accuracy: 93-94% (wheat, rice datasets); 85.4% (coffee)	Mobile-friendly transformer for on-field disease classification, enabling real-time mobile deployment.	Li et al., 2023
Fruits	orchards (multi-fruit)	CNN (YOLOv5) + GAN + Mean Teacher + Attention	RGB fruit images (different crops)	Accuracy: 87.2% (tomato), 89.9% (apple); +10.3% and +2.4% improvements *vs* existing methods	Significantly improved cross-fruit detection performance, effective transferacross fruit datasets.	(Guo et al., 2023)
Wheat	Outdoor field	Semi-supervised CNN Based Encoder–Decoder Domain adaptation	RGB images, video frames (wheat heads)	Strong generalization, scoring 80.7% Dice internally and 64.8% on external multi-country data across 18 domains.	Achieved robust segmentation performance across diverse field conditions with very limited labeling effort.	(Ghanbari et al., 2024)
Maize	Field-based HTPP maize plots	CNN segmentation model with bagging ensemble	High-resolution RGB images of maize plots)	mIoU = 0.70 (IoU_0_ = 0.67, IoU_1_ = 0.72; +7.7% *vs* single Deeplabv3+)	Improve segmentation accuracy under limited labeled data in field-based phenotyping.	(Zhang L. et al., 2024)
Black spruce	Field (Québec, Canada)	CNN, K-Means	Time-series PhenoCam images (RGB), Greenness Indices	IoU=0.9121 and an F1-score= 0.9235, (high accuracy)	PhenoAI automated phenology detection from near-surface cameras and multi-index time-series analysis.	(Kumar et al., 2025)
Wheat (spike detection)	GWHD2021 + Xinjiang field dataset (multi-country and site hybrid)	FEWheat-YOLO (CNN-based object detector (YOLOv11n))	UAV & smartphone field RGB images	R² = 0.941, MAE = 3.46, RMSE = 6.25	Model simplifications enable efficient, high-throughput wheat spike detection.	(Wu et al., 2025)

The controlled-environment phenotyping (left, e.g. greenhouse imaging systems with cameras on rails or conveyor belts) to field phenotyping (right, e.g. UAV drone surveys over crop plots).

### 3.1 Advances in deep learning for plant image analysis

#### 3.1.1 Deep learning reshaping plant image analysis

Over the last five years, DL has become the dominant approach for analyzing large plant image datasets, largely replacing earlier hand-crafted image analysis techniques. DL models can learn complex visual patterns of plant architecture, health, and development directly from data, reducing the need for human-designed features or heuristics (Murphy et al., 2024).

PhenoAI is an open-source Python framework for processing PhenoCam time-series images. It integrates quality control, vegetation segmentation, and phenological metrics extraction. In a black spruce case study in Quebec, it accurately detected key phenological stages, reducing manual work and improving monitoring efficiency (Kumar et al., 2025).

#### 3.1.2 CNNs: the foundation of modern plant image analysis

CNNs were one of the earliest DL architectures widely applied in plant phenotyping. Using large image datasets, researchers have trained CNNs to identify subtle phenotypic differences and to recognize plant structure (Ubbens et al., 2018). For example, CNN-based semantic segmentation models have been applied to partition images into meaningful classes, which distinguishing plant pixels from background or even segmenting individual leaves and organs in rosette plants and cereal canopies, thereby enabling precise measurement of traits such as leaf area, shape, and number; CNN models LeafNet have also been used for stress and disease phenotyping; by pairing classification networks with saliency maps, researchers can highlight which regions of a leaf or plant (e.g. edges, color patches) most contributed to a disease or drought stress prediction, aligning these regions with known symptoms and improving biological interpretability (Barré et al., 2017; Beikmohammadi et al., 2022).

In one early demonstration of DL’s potential, Pound et al. achieved over 97% accuracy in detecting and localizing plant organs from imagines Their deep CNN-based system could automatically find root tips and other root/shoot structures with great accuracy. The program can detect 12 of 14 QTLs that human experts had already found (Pound et al., 2017). This discovery showed that automated photo processing makes phenotyping easier, showing that phenotyping is moving toward data-driven era.

#### 3.1.3 Vision transformers: broadening the analytical scope

Vision Transformers (ViTs) have developed innovative techniques for processing plant images that exceed conventional models. Transformers show long-range interdependence and global spatial linkages in visuals by using self-attention methods. This feature makes it easier to see the general shape and context of a plant, while CNNs only look at small sections. ViTs are being used more and more to find plant diseases and for precision farming. They are good at working with different datasets and can even take good performance in hybrid models. Current research on transfer learning, model compression, and attention visualization is aiding in the reduction of data and computing requirements, underscoring their applicability for field utilization in crop monitoring and management (Mehdipour et al., 2025). ViT-based models can do plant phenotyping tasks with high accuracy and efficiency that is competitive. For example, a lightweight ViT model (PMVT) made for mobile devices was able to accurately classify plant diseases in the field (Li et al., 2023). Another method used convolutional features and transformer-based context integration (ST-CFI) to improve the ability to find diseases in leaves (Yu et al., 2025). Combined two ViT modules for extracting image features with a temporal transformer to model time-series data, then added seed information to estimate soybean production in Canadian farms. This method reduced the prediction error by more than 40% compared to the baseline and showed that seed traits play a key role, especially in identifying low-yield plots (Bi et al., 2023). Research indicates that Vision Transformers mitigate human and structural biases in picture analysis. This lets researchers examine at pictures more closely and get better features about plants with complicated traits.

#### 3.1.4 Overcoming data limitations through model fusion

A research study shown that employing a deep CNN segmentation network alongside an ensemble bagging technique significantly enhanced segmentation accuracy on a limited set of labeled crop images (Zhan et al., 2024). Deep 1D/2D CNNs were used to forecast yields in food-insecure areas based on normalized difference vegetation index (NDVI) and climate data. While the method transferred to Algeria (2002–2018), it performed worse than simple ML models and NDVI baselines, highlighting the limitations of small datasets for yield prediction (Sabo et al., 2023). As methods get better, they make outputs easier to understand, and help us understand phenotypic variance better.

### 3.2 Core computer vision tasks: segmentation, detection, and trait extraction

In controlled lab, acquired images are processed using semantic segmentation to identify specific plant organs (e.g., flower, leaf) and disease symptoms. Object detection algorithms further classify traits. UAV-based imaging enables large-scale trait extraction, and 3D reconstruction provides structural and morphological measurements.

**Figure 4:**
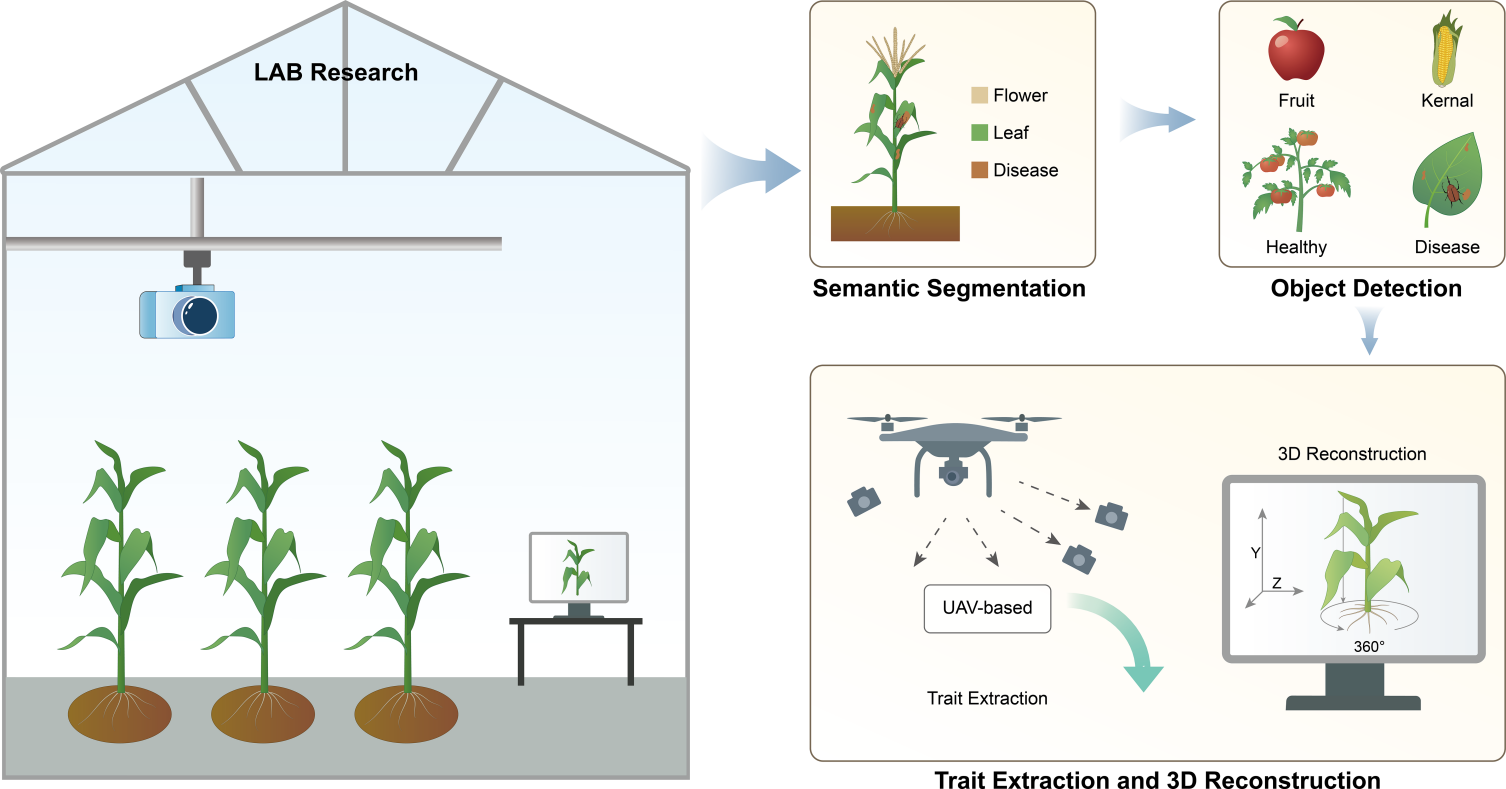
Controlled-environment Lab phenotyping. In controlled lab, acquired images are processed using semantic segmentation to identify specific plant organs (e.g., flower, leaf) and disease symptoms. Object detection algorithms further classify traits. UAV-based imaging enables large-scale trait extraction, and 3D reconstruction provides structural and morphological measurements.

For quantitative trait analysis, it based on basic computer vision tasks like segmentation, regression, and classification (Mostafa et al., 2023). Key vision tasks in plant phenotyping include semantic segmentation, object detection, and trait extraction through 3D reconstruction (**Figure 4**). AI has sped up progress in these areas.

#### 3.2.1 Semantic segmentation

Semantic Segmentation categorizes each pixel individually rather than considering the image as a whole (Osco et al., 2021b). Detailed pixel-level clarity lets you see the structure of plants in great detail, making it possible to precisely measure things like leaf area, canopy coverage, and organ morphology. Zenkl et al. employed a DeepLabV3+ model, trained on a variety of wheat photos, to effectively distinguish plants from the background across different genotypes (Zenkl et al., 2021). A UAV-enabled framework for cotton yield prediction based on cotton semantic segmentation using YOLO and SAM models (Reddy et al., 2024). Li et al. developed PSegNet, a framework for instance segmentation of 3D plant point clouds, enabling accurate organ identification combines voxel-based downsampling with multi-scale segmentation to provide better results across different plant species (Li et al., 2022). These instances demonstrate a shift towards sophisticated segmentation techniques that bring precision with AI.

#### 3.2.2 Object detection

Modern object recognition like Faster R-CNN and YOLO (Ren et al., 2017; Luo et al., 2024). have made it easier to do things like count fruits, find cereal spikes, and spot leaf defects. One of the biggest problems is generalizing to different species or varieties, since models that work well on one type of fruit may not work well on others that look different. To fix this problem, more and more people are using domain adaptation strategies.

Zhang et al. and Guo et al. studied fruit detection models to “fill the species gap”. Domain adaptation enhances the transferability of fruit identification models, facilitating cross-species application without the necessity for human labeling. When CycleGAN-based visual translation and pseudo-labeling were utilized, models trained on oranges did very well with apples and tomatoes (Zhang et al., 2021) Transfer performance has improved a lot because to cross-domain detection frameworks that leverage GANs and features to match images through knowledge distillation. To make sure that instance- and image-level representations are the same across domains, SSDA-YOLO uses YOLOv5, Mean Teacher distillation, and style transfer (Zhou et al., 2023). Adding contextual aggregation to improve global feature learning made it easier to transition models from datasets of oranges to datasets of tomatoes and apples. This showed that fruit recognition is more generalizable and efficient (Guo et al., 2023). FEWheat-YOLO (YOLOv11n) is a lightweight and efficient model achieved R² = 0.941 for Wheat Spike Detection (Wu et al., 2025). DomAda-FruitDet is a domain-adaptive model that doesn’t use anchors. It fills in the gaps between the foreground and background by using multi-scale prediction and adaptive sampling. This makes auto-labeling more precise and speeds up, thereby makes fruit recognition more general (Zhang W. et al., 2024).

These tactics, along with self-training, enabled the precise identification of fruits without manual labels. This form of domain adaptation is crucial for applying models to crops and in regions with limited data.

#### 3.2.3 Trait extraction and 3D reconstruction

Researchers that study plants have built a system that uses AI to look at drone pictures of crops from different angles. The technique automatically measures plant height and panicle length and creates realistic 3D crop canopy models. This method delivers images to the best ViT model based on clarity, noise, and blurriness. Segmentation accuracy and computing efficiency are higher than typical CNN approaches. Transformer designs can handle a variety of precision agricultural imaging situations, as shown by this modular design (Gopalan et al., 2025). To sum up the architectures used in agricultural research, we focus on two types of Transformer-based models: pure and hybrid (Xie et al., 2024). When put CNN and Transformer parts together, Transformers work from several viewpoints to display the overall shape of the plant, while CNNs gather small details like the margins of leaves and the textures of panicles. In 2023, Hu et al. introduced FOTCA, a hybrid plant disease detection model using adaptive Fourier neural operators and CNN-Transformer fusion. This design improves generalization and recognition by collecting local and global information (Hu et al., 2023).

In addition to 2D picture analysis, computational techniques are being utilized to rebuild plant morphology in three dimensions and to extract structural characteristics. Multi-view imagery and depth sensing make it possible to make 3D point clouds or volumetric models. Transformers are built into CNN-based stereo networks for plant research. UAV-based RS offers high-resolution monitoring that works well with yield data. DL models (CNN, LSTM, CNN-LSTM, ConvLSTM, 3D-CNN) were used to look at UAV RGB time series and meteorological data in Finland to estimate yield. The 3D-CNN achieved the highest accuracy, with an MAE of 219 kg/ha (5.5% MAPE) over a 15-week sequence and 293 kg/ha (7.2% MAPE) using early-season data (Nevavuori et al., 2020). Furthermore, EdgeMVSFormer, a transformer-based multi-view stereo method that reconstructs detailed 3D plant models from UAV images (Cheng et al., 2025). SegVoteNet represents a novel framework for 3D sorghum canopy phenotyping, by integrating UAV-based data acquisition, NeRF-based reconstruction, and DL-driven analysis. By integrating VoteNet and PointNet++ in a unified backbone, the model performs semantic segmentation and panicle detection directly from point clouds. This framework offers a scalable approach for canopy and panicle trait characterization in sorghum (James et al., 2025). Ninomiya (2022) analyzed high-throughput field phenotyping technologies, concentrating on diverse crop canopy characteristics, including plant height, coverage, biomass, stress indicators, and organ identification and enumeration. Improvements in photography, 3D reconstruction, sensor technologies, UAVs, and computational methods have made these observations possible (Ninomiya, 2022). Gaillard et al. devised a high-throughput voxel carving technique to recreate 3D models of sorghum plants from several RGB photos, facilitating the assessment of canopy features pertinent to light interception and genetic research (Gaillard et al., 2020). These 3D reconstructions make it possible to assess properties that are hard to see in 2D photos, such as plant structure, canopy volume, and branching patterns.

In short, DL-based segmentation, detection, and trait extraction dramatically increase the number of traits that can be measured from photos especially for varies situations.

### 3.3 Extending image-based phenotyping to real-world field conditions

**Figure 5:**
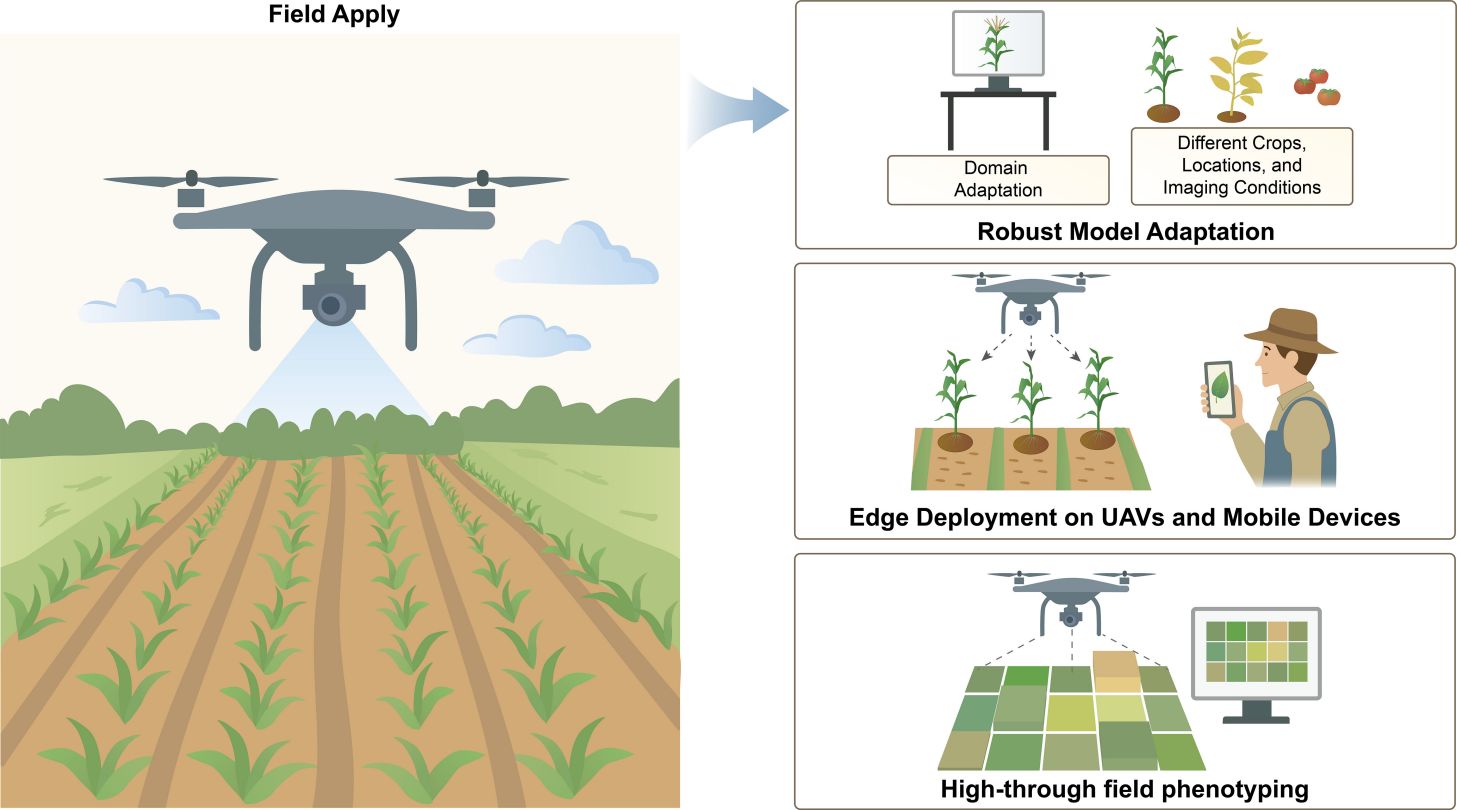
Image-based phenotyping to real-world field conditions. Domain adaptation allows models to generalize across crops and environments, while edge deployment supports real-time trait analysis. High-throughput field phenotyping allows rapid trait measurement in real-world agricultural environments.

One of the problems with image-based phenotyping is that it is hard to transfer approaches from controlled greenhouse to field settings, where changes in lighting, backdrop, weather, and plant structure can make models less reliable (**Figure 5**). Three primary research trajectories are tackling this deficiency.

Domain adaptation allows models to generalize across crops and environments, while edge deployment supports real-time trait analysis. High-throughput field phenotyping allows rapid trait measurement in real-world agricultural environments.

#### 3.3.1 Robust model adaptation

Models that work well in one situation (like greenhouse photos of plants that all develop the same way) frequently don’t work well in another (like field plots with changing conditions). Recent research has investigated semi-self-supervised domain adaptation, utilizing little manual annotation alongside video-derived data and neural encoder-decoder architectures to produce substantial training datasets. These methods have shown good results in a variety of fields, showing that they could make DL applications in agriculture more stable and scalable (Ghanbari et al., 2024). As noted in the last section, researchers are now using domain adaptation and intense data augmentation to make models more robust.

For example, large annotated field image datasets such as the Global Wheat Head Detection (GWHD) dataset (David et al., 2020) have been established to facilitate training of wheat spike detection models across diverse genotypes and locations. Exposure to such diverse training data helps models learn more generalized representations. Techniques such as adversarial domain adaptation and synthetic data generation are also applied. One study proposed a semi-self-supervised CNN with probabilistic diffusion, requiring minimal manual labels and achieving robust crop image segmentation across variable field conditions (weather, lighting changes) (Ghanbari et al., 2024). The focus is on careful dataset design and curation. Training that covers a wide range of environmental circumstances makes models more stable when they are used in the real world. Transfer learning through fine-tuning on small datasets is commonly used to quickly adapt models to new crops or sites without having to relabel a lot of data.

Recent developments have investigated the application of Generative Adversarial Networks (GANs) to reduce dependence on extensive annotated datasets. Varela et al. introduced the Efficiently Supervised GAN (ESGAN) framework, which integrates extensive amounts of unlabeled UAV footage with a limited collection of annotated samples to precisely categorize heading phases in Miscanthus breeding populations (Varela et al., 2025). ESGAN was able to equal the accuracy of fully supervised models using only 1% of the labeled data, which made labeling much less of a chore. Its generator-discriminator design allows it to work well under changing field circumstances, making it a scalable solution for complicated agricultural situations (Depaepe, 2025).

#### 3.3.2 Edge deployment on mobile devices and UAVs

To reach this goal, models need to be quickly put on edge devices like drones, robots, and mobile phones without using the cloud. Lightweight architectures and model compression methods like pruning, quantization, knowledge distillation, and deep compression have made it possible to run on less powerful hardware by using less memory and computing resources. This method keeps accuracy while cutting the network’s storage needs by more than 30 times. Tests on AlexNet and VGG-16 demonstrated that compressed models can fit in memory on the chip, which speeds things up and uses less power. This enables you employ complicated networks on mobile and embedded devices (Han et al., 2016). MobileNetV3 is a new and efficient CNN design that uses hardware-aware neural architecture search, the NetAdapt algorithm, and other advancements to the architecture. This leads to MobileNetV3-Large for apps that need a lot of resources and MobileNetV3-Small for apps that don’t. These models make it faster and more accurate to classify, detect, and segment images semantically. MobileNetV3-Large and the LR-ASPP decoder are superior than MobileNetV2 and have shorter lag time (Howard et al., 2019).

DL and smartphones work together to make it feasible to find crop illnesses on a big scale. A revolutionary study trained a CNN on 54,306 leaf pictures from 14 species and 26 diseases, achieving 99.35% accuracy and demonstrating the potential for large-scale smartphone-based detection (Mohanty et al., 2016).

Recent UAV phenotyping research has focused on lightweight hardware suitable for field deployment. Improvements have made it possible to process data in real time, which means that data doesn’t have to be moved around as often. A number of studies show that optimized embedded models can be just as accurate as bigger cloud-based systems. This means that they may be able to keep working well in the field.

Measuring features like leaf color, leaf area index (LAI), chlorophyll content, biomass, and yield by hand is time-consuming and not very effective. UAV remote sensing platforms (UAV-RSPs) with different sensors, on the other hand, now offer a flexible, fast, and non-destructive way to do large-scale phenotyping (Yang et al., 2017). Later, Osco and his team did 232 studies utilizing pictures taken by UAVs. They looked into a lot of different application areas, sensor integration, and ways to classify and regress. Their experiment showed how well-made models can help in monitoring crops and phenotyping in the field (Osco et al., 2021a). Guo et al. examined recent developments in UAS-based plant phenotyping, emphasizing its cost-effectiveness, versatility, and potential to integrate many sensors in settings (Guo et al., 2021).

To automatically look at canopy images, CimageA is a software system that can grow and uses machine vision and ML. It helps quickly and accurately determine phenotypes such as LAI, canopy coverage, and plant height (Fu et al., 2024). To strike a balance between speed and precision, there are two optimized models: MobileNetV2-UNet and FFB-BiSeNetV2. TensorRT was used to optimization to execute them on Jetson AGX Xavier. MobileNetV2-UNet reduced the number of parameters and calculations while speeding up inference. FFB-BiSeNetV2 had a mean Intersection over Union (IoU) of 80.28%. Both models worked in real time (more than 40 frames per second), showing that UAV-based image analysis is a good way to manage weeds and safeguard plants (Lan et al., 2021).

The lightweight model design, embedded hardware capability, and UAV sensing platforms are making the next generation of scalable solutions for plant phenotyping.

#### 3.3.3 High-throughput field phenotyping

Drones with RGB or multispectral sensors quickly take pictures of field plots and get traits faster in the sectors of precision agriculture and breeding. Future breeding advancements necessitate the development of cost-effective, field-deployed high-throughput phenotyping devices (HTPPs). They need to combine large-scale, non-invasive sensing with automated environmental monitoring and fast ways to collect, score, and analyze data (Araus and Cairns, 2014).

A detailed study looked at how Unmanned aerial system (UAS)-based high-throughput phenotyping can be used in breeding programs. It covered tools, methods, direct trait measurement, predictive breeding, QTL finding, and the ability to speed up genetic gain (Khuimphukhieo and da Silva, 2025). Moreover, DL has enhanced both the throughput and accuracy of these UAV phenotyping pipelines. Yang et al. developed a UAV image analysis method that accurately extracts plot boundaries from field trial images, greatly speeding up trait measurements (Yang et al., 2021). Tang et al. demonstrated automated plot delineation and biomass estimation in alfalfa field trials. Using canopy area and plant height features extracted from images, the model achieved an R² of about 0.6 for biomass prediction without manual input. Multitask DL frameworks have also been applied to predict multiple traits from the same images (Tang et al., 2021). UAV-based multisensory data fusion and GeoAI (a combination of geospatial and artificial intelligence research) was designed for high-throughput maize phenotyping. UAVs equipped with hyperspectral, thermal, and LiDAR sensors were used to predict eight yield- and nitrogen-related traits. Extended NDSI analysis, classical ML models (SVM, RF), and a multitask CNN were compared. Integrating hyperspectral and LiDAR data improved prediction accuracy, and the multitask CNN achieved performance comparable to or better than single-task models, demonstrating the potential of GeoAI and sensor fusion for field-scale trait prediction (Nguyen et al., 2023).

Tausen et al. developed Greenotyper, a low-cost, distributed HTP platform using 180 Raspberry Pi cameras to monitor 1,800 clover plants and generate over 355,000 images in one experiment. A U-Net based pipeline achieved ~98% plant localization accuracy and 95% segmentation accuracy. All images were processed within one day with 96% system uptime, demonstrating an efficient and scalable alternative to traditional phenotyping platforms (Tausen et al., 2020). MtCro is a multi-task framework that models associated phenotypes together in a shared parameter space. It did better than DNNGP and SoyDNGP on big datasets of wheat and maize, making predictions up to 9% more accurate and making predictions for several phenotypes better (Chao et al., 2025).

In the end, the outcome is a phenotyping pipeline that can be scaled up and can fly a drone over a field and automatically extract yield-relevant features from each plot within minutes of data acquisition. This gives plant breeders data-driven insights that are almost real-time for making decisions about which plants to breed. It let crop researchers run tests on thousands of genotypes in different settings, helping farmers on their fields manage with accuracy.

### 3.4 Open Science platform in plant phenotyping

As image-based phenotyping grows, data needs to be shared and made more open. Open platforms let users add new features or functions, which encourages collaboration in community. Tools like PlantCV (Gehan et al., 2017) and Image Harvest (Knecht et al., 2016) offer researchers standardized image analysis methods that are accessible at no cost. Deep Plant Phenomics offers pre-trained neural networks for plant phenotyping and enables researchers to tailor the models to their particular requirements. In Arabidopsis thaliana, it has showed promising mutant classification and age regression with high counting accuracy (Ubbens and Stavness, 2017). Furthermore, the publication of the Global Wheat Head Detection Dataset (GWHD) was a collaborative initiative aimed at establishing a substantial and varied standard for head detection algorithms (David et al., 2020).

Alongside software, there is an initiative for enhanced data governance and adherence to Findable, Accessible, Interoperable, and Reusable (FAIR) standards (Papoutsoglou et al., 2023).

Common benchmark datasets improve algorithm development and allow research organizations to compare results. Researchers are standardizing data to make collaboration easier. Researchers increasingly share code and models, making results easier to replicate and strengthening trust in open science.

## 4 Remote sensing for yield and trait prediction

Beyond image-based phenotyping, recent analytical methods highlight data from several sources to better describe environmental aspects.

Forecasting agricultural yield is a plant area priority. Traditional agronomic methods use experience criteria and provide only basic biological process representations, making it difficult to understand agricultural systems. Many methods fail to capture the complex genotype×environment (G×E) interactions that impact agricultural outcomes. In recent years, AI has shown a remarkable ability to find patterns in large, heterogeneous datasets, complementing traditional models. Recent progress in HTP has enabled multi-source frameworks that integrate different data for yield and trait prediction. These may include time-series satellite imagery, weather data, soil maps, management records, and even genomic information about crop varieties. The data are diverse in nature (spatial, temporal), and accordingly, flexible AI architectures are employed to fuse them. At the same time, challenges remain in terms of model generalizability across environments and seasons when predictions are used to inform agricultural decisions or policy.

**Figure 6:**
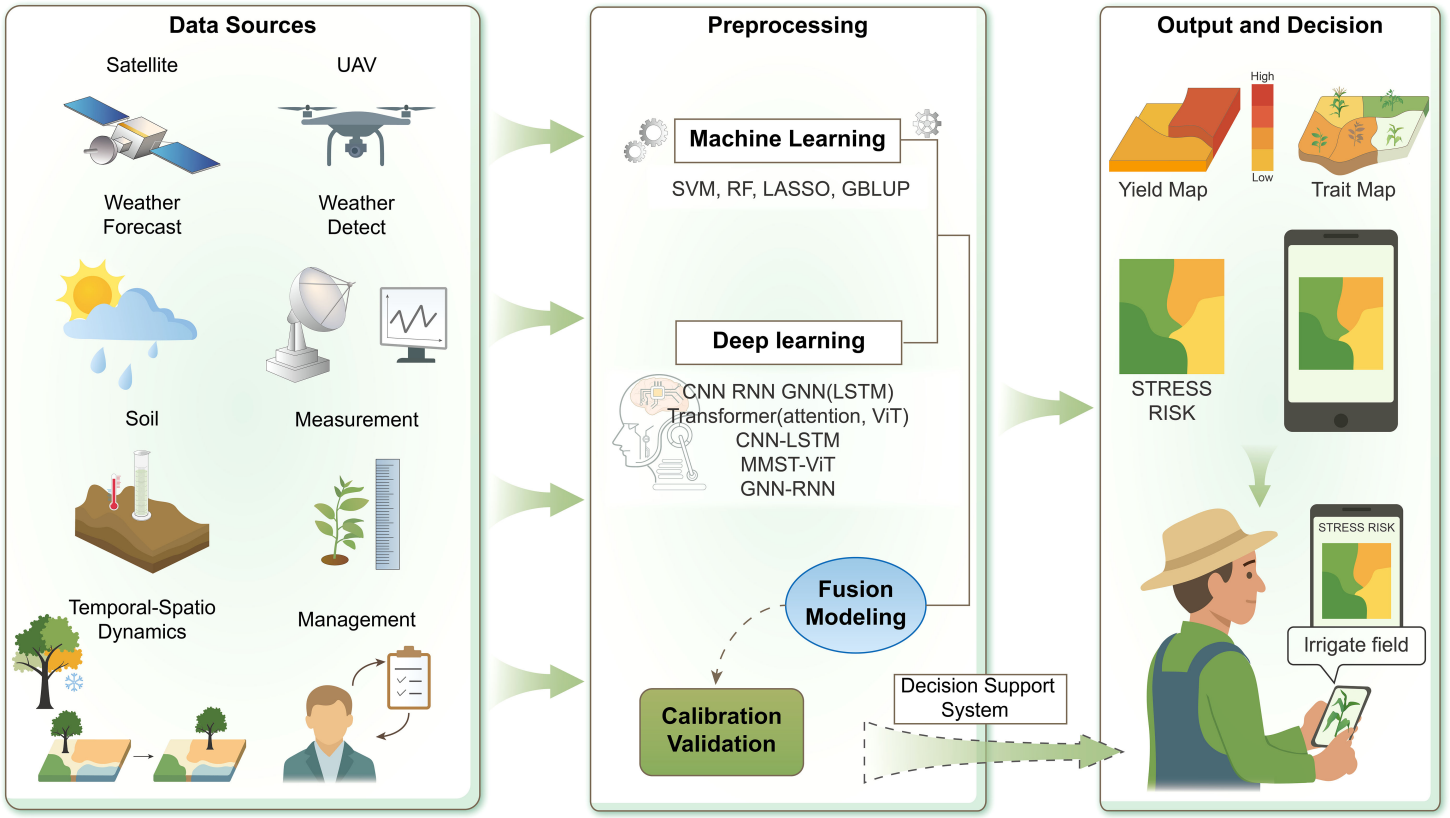
AI-enabled workflow for crop monitoring and decision support. Multisource environmental, phenotypic, and management data are processed using machine learning and deep learning models. Model fusion and calibration produce spatial yield and trait maps, enabling real-time, data-driven agricultural management.

In this section, we will outline how multi-source data are integrated for crop prediction, the algorithmic advances for spatial-temporal modeling, and key applications from farm to global scales (**Figure 6**; **Table 3**).

**Table 3:** Representative AI-based models for crop yield and trait forecasting.

Crop	Region	AI model	Data type	Performance metrics	Key findings	Reference
Wheat (winter)	South Asia (district scale)	LSTM-based model (with integrated climate & NDVI)	MODIS NDVI time-series + climate variables	R² = 0.78–0.82(depending on district/year)	Enabling accurate district-level yield predictions in South Asia without field surveys using only satellite NDVI sequences and climate data.	(Ashfaq et al., 2025a)
Wheat (winter)	Southern Pakistan (district scale)	DeepAgroNet (multi-branch CNN-RNN-ANN ensemble)	Satellite imagery + weather time-series + soil data	R² = 0.77; 98% accuracy one month before harvest; <10% error	Dramatically improving early forecasting for Pakistan’s wheat belt.	(Ashfaq et al., 2025b)
Wheat (winter)	Henan, China (provincial)	CNN + BiLSTM (Bayesian optimized; “BCBL” model)	Satellite indices (EVI, LAI, SIF) + climate	R² = 0.81; RMSE = 617 kg/ha; MRE = 7.14%	Novel satellite data with traditional indices and weather data for yield prediction. Stable yield estimates 25 days before harvest.	(Zhang et al., 2024)
Maize (field scale)	U.S. Midwest (Corn Belt)	DeepCropNet (Attention LSTM + multitask learning)	Historical yield + climate data (1981–2016)	RMSE = 0.82 Mg/ha (*vs* 1.14 LASSO and 1.15 RF)	Effectively captured temporal patterns like weather trends, boosting accuracy.	(Lin et al., 2020)
Crops (mixed)	Indian	FedGAvg (FL-AGRN (Federated Attention GNN + RNN))	Agricultural Crop Yield in Indian States Dataset (19,524 data points)	R² = 0.99; MAE = 1.23; RMSE = 1.45	This demonstrated scalable, confidential yield forecasting across regions.	(Nirosha and Vennila, 2025a)
Muitiple Crops	Multi-region (trial plots) farms located in Ames, Iowa	Agri-GNN (GraphSAGE-based neural network)	Vegetation indices + genotype + time + location	RMSE=4.565, MAE = 3.590, R 2 = 0.876	More accurate plot-level yield estimates than KNN methods and underscoring GNNs in breeding.	(Gupta and Singh, 2023)
Corn, cotton, soybean, winter wheat	Mississippi, Louisiana, Iowa, Illinois	MMST-ViT (Transformer)	Sentinel-2 imagery, HRRR meteorological data, USDA crop data	Corn RMSE = 10.5, Corr=0.918; Soybean RMSE = 3.9, Corr=0.900;	The model also demonstrates robust behavior by enabling reliable one-year-ahead yield prediction	(Lin et al., 2023)
Soybean	USA	CNN + LSTM (hybrid model)	MODIS NDVI imagery + climate, via Google Earth Engine	End-of-season RMSE = 329.53	Improved both in-season and end-of-season county yield predictions.	(Sun et al., 2019)
Mango	Australia (orchard blocks)	Machine Learning (time-series regression)	8-year yield history + satellite VIs + climate	Block-level 2021 (MAE = 2.9 t/ha) and RF model (farm = 3.7%, block (NMAE) = 33.6% for 2021)	Maintaining reliable predictions for orchard yields without fruit counting, greatly cut down on labor.	(Torgbor et al., 2023)
Maize	Missouri, USA (farm trial)	Machine Learning (for best SVR/KNN models))	UAV multispectral imagery (V6 & R5) → spectral bands + 26 Vis	Fallow(SVR) V6:R² = 0.84, RMSE = 0.69 Mg/ha R5:R² = 0.83, RMSE = 1.05 Mg/ha	Demonstrated accurate yield estimation with minimal training data of Simpler ML models (SVR, k-NN) slightly outperformed deep networks.	(Kumar et al., 2025)
Cotton	Texas, USA (field rows)	YOLOv8 + SAM (object detection & segmentation)	UAV RGB images (open-canopy cotton bolls)	R² = 0.913, Low MAE	Provides a robust, rapid yield estimation linking image segmentation to cotton yield with high confidence.	(Reddy et al., 2024)
Multi-crop (10)	Global (cross-continent)	Machine Learning	Historical yields + climate/soil/management (global dataset)	R² > 0.8 prediction accuracy	Machine learning can capture broad G×E patterns at scale and improve global yield projections.	Neumann and Furbank, 2021
Maize (multimodal HTP)	Illinois, USA (experimental corn field)	Multitask CNN (GeoAI sensor fusion framework)	UAV hyperspectral + thermal + LiDAR data	R² up to ~0.85 for N traits; ~0.5–0.6 for biomass traits; ~0.34 for grain density (multi-task CNN comparable or slightly better than mono-task and RF/SVR).	The sensor fusion improved prediction robustness, highlighting the value of multimodal data for field-scale yield trait estimation.	(Nguyen et al., 2023)
Wheat (genomic G×E)	Multi-country (breeding trials)	DNNGP	Genomic markers + transcriptomics (for maize) + multi-environment phenotypes	+9% accuracy *vs* prior GP methods	Accelerating selection of high-yield lines under varying conditions.	(Wang et al., 2023)

Multisource environmental, phenotypic, and management data are processed using machine learning and deep learning models. Model fusion and calibration produce spatial yield and trait maps, enabling real-time, data-driven agricultural management.

### 4.1 Diverse data sources and multi-source inputs

Crop yields are influenced by multiple factors: weather throughout the season (rainfall, temperature extremes, solar radiation), soil properties (nutrient levels, texture, water-holding capacity, PH), and management (planting date, fertilization, irrigation). A typical multi-source yield prediction pipeline might take as input a sequence of satellite or UAV images (capturing the crop’s canopy development via vegetation indices), a time-series of weather variables, static soil and topography maps, and categorical variables like crop type or cultivar. AI models extract these disparate inputs that might be hard to specify in explicit models then learns to associate patterns in these various inputs with final yield.

#### 4.1.1 Machine learning approaches for prediction

Researchers have shown that ensembles of ML models can effectively map and predict yields by learning from past yield statistics and environmental data. For example, Neumann and Furbank (2021) compiled a continent-wide yield database covering ten major crops and extensive environmental and management variables. Using an ensemble of ML methods, they achieved cross-continental yield prediction accuracies exceeding R² = 0.8 for some crops, despite the high environmental and management diversity (Newman and Furbank, 2021). The progress in using remote sensing and ML for detecting and managing Invasive Plant Species (IPS)-High-resolution imagery from UAVs, satellites, and hyperspectral sensors, combined with ML approaches has improved mapping accuracy and ecological assessment of IPS (Zaka and Samat, 2024). Integrating technologies such as LiDAR and cross-disciplinary modeling can improve monitoring and conservation under climate and urbanization pressures. UAV-derived vegetation indices combined with ML have been used for accurate corn yield prediction with limited training data. Using multispectral data from V6 and R5 stages under different soil treatments, support vector regression (SVR) and k-nearest neighbor (KNN) performed best, with red-edge and chlorophyll indices as key predictors (Kumar et al., 2023). Such studies indicates when enough historical data are available, ML approaches can capture yield determinants across climate zones and farming systems in ways.

At local scales, researchers use freely available satellite imagery and weather data to estimate yields without relying on additional field surveys. In mango orchards, Torgbor et al. developed a time-series modeling framework for yield forecasting without in-field fruit counting. Using eight years of yield data from 51 orchard blocks, together with remotely sensed vegetation indices and climatic variables, ML models (e.g., RF, SVM) achieved accurate yield predictions while reducing the need for extensive ground sampling (Torgbor et al., 2023). Genomic prediction models use genome-wide DNA markers (e.g. SNPs) to predict the breeding values of individuals termed genomic estimated breeding values (GEBVs) without extensive field testing. These ML models have been widely adopted in plant breeding and can predict cultivar performance in different environments by accounting for G×E interactions (Crossa et al., 2025b).

A wide range of ML algorithms have been applied to implement models for G×E: such as GBLUP, a linear mixed model using the G matrix (Nishio and Satoh, 2015). GBLUP captures additive genetic effects and is robust for large marker sets. Bayesian genomic models Methods like Bayes A, Bayes B, Bayes C, Bayesian LASSO, etc. potentially improving prediction when some QTL have large effects (Shi et al., 2021). Reproducing Kernel Hilbert Space (RKHS) methods have been widely utilized in plant breeding for their capacity to model non-linear genetic architectures, including epistatic and G×E interactions. As one of the earliest ML approaches adopted in this field, RKHS regression projects marker genotype data into a high-dimensional function space via kernel function (Gianola and van Kaam, 2008; De Los Campos et al., 2010). RF had been used for genomic predictions and can handle many predictors by averaging over many tree models. It can model G×E by including environment indicators or interactions as features (Stephan et al., 2015). SVM find optimal separating hyperplanes and can be used with kernels for regression (SVR). They have been tested on genomic datasets to capture non-linear genotype-phenotype relationships for very large datasets for genomic prediction (Montesinos López et al., 2022). All kinds of ML approach are highly scalable, since satellite data and gridded weather are available, making it feasible to extend yield prediction to regions where field surveys are impractical.

#### 4.1.2 Combined deep learning for seasonal yield prediction

Neural networks, such as Multi-Layer Perceptrons (MLPs) and CNNs, can automatically capture complex feature interactions, particularly when handling large-scale datasets. As extensively reviewed, DL methods are capable of integrating diverse data sources, including image-based HTP and genomic markers to enhance G×E predictions. Furthermore, emerging architectures like the DNN Genomic Prediction (DNNGP) framework set a precedent for fusing multi-omics data (Wang et al., 2023). A pioneering example of a multi-source architecture for yield prediction is DeepAgroNet, which employs a multi-branch neural network to handle different data types: one branch is a CNN that processes spatial features from satellite images, another branch is Long Short-Term Memory (LSTM) that captures temporal patterns in sequential weather data, and a third branch is a feed-forward network for static variables. By merging these branches, the model can acquire a unified depiction of the environment and agricultural circumstances for different place and period (Ashfaq et al., 2025b).

The FL-AGRN model predicts crop yields by integrating attention-based GNNs and RNNs inside a FL architecture (Nirosha and Vennila, 2025a). A GNN-RNN framework for predicting crop yields across the country clearly considered both geographical and temporal correlations. The model included data from more than 2,000 U.S. counties from 1981 to 2019. It did better than linear, tree-based, and DL baselines (Fan et al., 2022). The model attained great accuracy (Mean Absolute Error (MAE) = 1.23, Root Mean Square Error (RMSE) = 1.45, R² = 0.99) through distributed training with a Federated Gaussian Average aggregator and meticulous data pretreatment, all while ensuring data confidentiality and scalability. It did better than traditional methods and made the yield forecasts more accurate (Nirosha and Vennila, 2025b). The Agri-GNN also established to simulate crop yield by explicitly addressing both spatial and genotypic interactions among farming plots. Agri-GNN is built on a GraphSAGE architecture and combines vegetation indices, genotype, time, and location data. It works better than ML methods, showing that graph-based neural architectures have a lot of potential for agriculture (Gupta and Singh, 2023).

Temporal dynamics are especially important in yield formation: crops progress through growth stages, and mid-season indicators (e.g. a severe drought during the flowering stage, or a period of low NDVI for stress) strongly influence final yield. AI models enabling early detection. Recurrent neural networks like LSTM and GRU are natural choices to handle time-series input. In winter wheat yield prediction, researchers showed that using multi-month NDVI time series as input to an LSTM improved yield estimates compared with single-image or cumulative index approaches. The sequential data enabled the model to account for early growth patterns and stress (Sharma et al., 2020; Zhang et al., 2024). Moreover, LSTM-based models can integrate information across the entire season (from emergence to grain filling) A recent study combined meta-transformer for multi-source feature fusion with temporal graph neural networks for sequential modeling, achieving nearly 97% accuracy on the EPFL dataset and outperforming LSTM, CNN, and standard Transformer baselines (Sarkar et al., 2024). Furthermore, convolutional temporal networks and even 1D CNNs have been used to extract features from weather time-series for yield prediction, summer crops using Landsat EVI time series, which can outperform RNN, RF, and SVM in accuracy and F1 score (Saha et al., 2025).

Transformer-based models, which excel at long-sequence tasks have been applied to multi-year yield forecasting. Transformer’s attention mechanism can in principle learn to weight the most critical periods (e.g., weeks of drought stress) more heavily. In one study, a transformer model outperformed LSTMs for early-season county-level yield prediction in the U.S., likely due to its ability to flexibly incorporate historical information and early-season imagery with varying importance; Multi-Modal Spatial-Temporal Vision Transformer (MMST-ViT), which integrates satellite imagery with meteorological data through multi-modal, spatial, and temporal transformer modules. This framework accounts for both short-term weather variability and long-term climate influences. Experiments conducted in more than 200 U.S. counties showed that MMST-ViT achieved better performance comparable methods on multiple evaluation metrics (Lin et al., 2023). Najjar et al. submitted a paper to ICLR 2025 that was later withdrawn. It looked at how easy it is to explain Multimodal learning frameworks for predicting crop yields. The research utilized Transformer-based architectures to amalgamate many modalities, including satellite imagery and environmental data, while implementing self-attention and feature attribution techniques to improve interpretability and perhaps yield more robust predictions (Najjar et al., 2025).

### 4.2 Modeling temporal-spatio dynamics

Combining both spatial and temporal dimensions is a key problem in crop modeling. Topography, soil heterogeneity, microclimates, the timing of stresses (drought, heat, pest outbreaks), and the path of crop growth (e.g., rapid early growth vs. slow start) can all affect how crops grow and how much they yield.

In traffic field previous studies of TedTrajRec, is proposed to improve recovery performance. PD-GNN is employed to model periodic and topologically aware traffic patterns before, while TedFormer, a time-aware Transformer with neural ordinary differential equations, effectively learns temporal dependencies from irregularly sampled data (Sun et al., 2025). In AI-Plant field, AI-based approaches address spatial heterogeneity by incorporating remote sensing at multiple resolutions, such as combining coarse satellite data for regional climate context with high-resolution drone images for fine-grained within-field features. AI can extract spatial features (textures, patterns) that correlate with crop health, while graphical AI applied to geospatial grids can capture interactions between neighboring areas (e.g. disease spread or irrigation patterns).

#### 4.2.1 Spatio-temporal dynamics

Early spatio-temporal rice mapping mainly used phenology- or feature-based analysis of optical and SAR time-series data with thresholding and rule-based classification. A phenology-based algorithm (ILMP) combining Landsat and Moderate-Resolution Imaging Spectroradiometer (MODIS) achieved over 93% accuracy in Nanchang (2015), performing better than NLCD in fragmented cropland (Ding et al., 2020). The PKI method using Sentinel-1 SAR time series achieved 97.99% accuracy in paddy rice mapping, outperforming phenology-based methods and enabling large-scale application (Lin et al., 2024).

Early rule-based methods laid the basis for DL approaches that capture complex spatiotemporal signals. Crop mapping has since shifted from phenology analysis to data-driven feature extraction. Lightweight CNN combined with parcel-based image analysis was developed for crop classification using Sentinel-2 time-series imagery. Applied to two regions in Türkiye, the model achieved overall accuracies of 89.3% and 88.3%, outperforming VGG-16, ResNet-50, and U-Net, and demonstrating its cost-efficient crop mapping (Altun and Turker, 2025). Another approach for spatio-temporal modeling is the use of encoder-decoder frameworks that take sequences of input images (or weather maps) and predict sequences of outputs (like maps of predicted yield). CNN capturing spatial features and LSTM modeling phenological dynamics. A recent soybean study proposed a CNN-LSTM model for county-level soybean yield prediction across CONUS, integrating weather and MODIS data via Google Earth Engine. The hybrid model outperformed alone CNN or LSTM approaches for both in-season and end-of-season prediction, showing promise for broader crop applications (Sun et al., 2019).

An attention-based Geo-CBAM-CNN model was developed for crop classification using Sentinel-2 time-series imagery. By integrating geographic information into an attention module, it effectively mitigated spatial heterogeneity and enhanced spectral-spatial feature extraction. Validated across multiple U.S. regions, it achieved 97.82% overall accuracy across multiple U.S. regions and showed strong spatial adaptability for large-scale applications (Wang et al., 2021). To address the challenges of nonlinear spatiotemporal dependencies in yield prediction, a knowledge-guided Spatial-Temporal Attention Graph Network (KSTAGE) was proposed. By integrating spectral features with prior knowledge through temporal attention and spatial graph modeling, KSTAGE achieved significant improvements over baseline models in county-level yield prediction tasks both in China and the U.S (Qiao et al., 2023). The CNN- Self-Attention based model effectively captures fine spatial and long-term temporal patterns from high-resolution imagery. DL framework SepHRNet combining HRNet and Self-Attention was proposed for crop mapping, achieving 97.5% accuracy and outperforming state-of-the-art models on the Zuericrop dataset (Goyal et al., 2025). For large-scale corn yield estimation, a deep spatiotemporal framework (DeepCropNet, DCN) was developed that combines Attention-Based LSTM (RNN) for temporal features and multitask learning for spatial features. Applied to U.S. Corn Belt data (1981–2016), DCN outperformed LASSO and RF (RMSE = 0.82 vs. 1.14 and 1.05 Mg ha^-^¹), effectively capturing temporal effects and patterns (Lin et al., 2020).

Both Cropformer and AgriFM are Transformer-based models, with Cropformer focusing on multi-scenario crop classification and AgriFM extending to large-scale, multi-source spatiotemporal crop mapping. Cropformer, a self-supervised and fine-tuned Transformer-based model, enables accurate and transferable multi-scenario crop classification from time-series remote sensing data (Wang et al., 2025). AgriFM, a multi-source remote sensing foundation model designed for crop mapping through unified multi-scale spatiotemporal feature extraction. Built on a modified Swin Transformer and pre-trained on over 25 million samples from MODIS, Landsat, and Sentinel-2, it supports diverse tasks such as cropland mapping, boundary delineation, and early-season crop classification. It outperforms remote sensing foundation models (RSFMs), showing strong scalability and adaptability (Li et al., 2025).

The new trend is Spatial-Temporal (ST) is GNN, by structuring the data as a graph, these approaches can incorporate domain knowledge (e.g. adjacency or shared water sources) and have the model learn how information flows in both space and time. The Spatial-Temporal Synchronous Graph Convolutional Network (STSGCN) was proposed to address complex localized spatiotemporal correlations and heterogeneities in network data forecasting (Song et al., 2020). A study introduced a hyperspectral maize nitrogen prediction model that combines a dynamic spectral-spatiotemporal attention mechanism with a Graph Neural Network (GNN). The technique attained great accuracy (R² = 0.96) and surpassed models like SVM, RF, ResNet, and ViT, exhibiting strong adaptability throughout developmental stages and geographical contexts (Lu et al., 2025).

These modifications indicate employing advanced AI techniques brings a transition from rule-based phenological mapping to integrated spatiotemporal modeling.

#### 4.2.2 Operational deployment and decision support

Remote sensing and spatially explicit yield prediction can help in making decisions about farming and policy. Satellite-based AI models allow the United Nations (UN) Food and Agriculture Organization (FAO) to monitor drought’s effects on crops worldwide in real time. The Agricultural Stress Index System (ASIS) from the FAO combines digital innovation with decades of satellite data, such as long-term AVHRR-derived vegetation health indicators. ASIS helps with anticipatory action, crop insurance, and drought management both on a national and regional scale (Van Hoolst et al., 2016; Rojas, 2021).

### 4.3 Representative applications with case studies

From research to practice, data-driven yield and trait prediction has proven effective for crop management. Some of the important uses are:

#### 4.3.1 Field trial analytics

Field trials at multiple sites generate thousands of experimental crop genotypes. AI models are utilized to guess important qualities like yield and drought tolerance, as well as meteorological conditions and soil data from each of the location. A study on maize used multitask DL to predict multiple traits from UAV imagery simultaneously, improving the selection of promising hybrids GNN have also been employed to leverage genetic relatedness information alongside environment data, enhancing the prediction of genotype performance across trial sites (Zhou et al., 2025).

#### 4.3.2 Regional yield forecasts

Prior to harvest, governments and commodity markets seek reliable yield forecasts. AI models ingest satellite time-series for crop cover and weather data to forecast yields at district or county levels. Ashfaq et al. demonstrated forecast for wheat in South Asia using an LSTM on NDVI sequences (Ashfaq et al., 2025a), and similar approaches have been tested for corn and soy in the U.S. Corn Belt (Han et al., 2024). These forecasts can inform logistics (e.g. storage and transportation needs) and market pricing, therefore predict early interventions.

#### 4.3.3 Stress and resilience trait prediction

AI can predict traits like drought tolerance, disease outbreak, or nutritional deficiencies using remote sensing data, in addition to yield. Models have been taught to forecast drought stress indices for each pixel in a satellite image, which shows where crops are having trouble getting enough water (Desloires et al., 2024). ML also forecasts when pests and diseases will happen by noticing small changes in the canopy before they happen, which allows for early management actions.

In these applications and case studies, typical problems include making models work in novel situations and making forecasts easier for end users to understand. SHAP (SHapley Additive exPlanations) is one of the methods used to figure out which elements had the biggest effect on a prediction (Lundberg and Lee, 2017; Crane-Droesch, 2018). With advancements in hardware and data infrastructure, the obstacles to large-scale deployment of these models are diminishing.

### 4.4 Field-based AI-enabled yield forecasting

Nowadays, a growing body of evidence demonstrates that AI methods substantially improve yield forecasting across remote-sensing and environmental data streams. Among these approaches, both ML and DL methods have shown clear advantages in capturing complex spatial and temporal patterns for yield forecasting.

#### 4.4.1 Machine learning based yield forecasting

Accurate yield estimations are increasingly rely on remote sensing, and the growing data volume has made machine learning essential for handling complex, nonlinear information and improving prediction accuracy in modern agricultural systems (Chlingaryan et al., 2018). A global assessment across wheat, maize, and potato showed that RF provide notably higher yield-prediction accuracy than linear regression, with RF achieving RMSE of 6-14% compared to 14-49% for linear models. Moreover, yield data was from various sources and regions for model training and testing: gridded global wheat grain yield, maize grain yield from US counties over thirty years, and potato tuber/maize silage yield from the northeastern seaboard region for large-scale climate-driven yield forecasting (Jeong et al., 2016). ML ensemble to forecast maize yields across three U.S. Corn Belt states using both complete and partial in-season weather information. Weighted and average ensemble models achieved high accuracy, and were able to generate reliable early-season forecasts as early as June 1, RRMSE of 9.2% (Shahhosseini et al., 2020).

#### 4.4.2 Deep learning based yield forecasting

Using multi-temporal MODIS surface reflectance data on satellite across major U.S. soybean-producing states, You et al. developed a deep Gaussian process framework that reduced county-level RMSE by approximately 30% relative to the best traditional remote-sensing models and delivered earlier-season forecasts with steadily improving skill. When aggregated to the national level, the model also achieved about 15% lower MAPE than USDA survey-based estimates in August and September, demonstrating competitive performance well before harvest (Jiaxuan et al., 2017). A DL framework combining CNN and RNN architectures has been used to forecast corn and soybean yields across the U.S. Corn Belt, integrating environmental and management time-series data. The CNN-RNN model achieved notably low errors (8-9% RMSE of average yield), outperforming RF and LASSO, and demonstrated strong generalization to unseen environments. Its design enables the extraction of temporal environmental signals allowing attribution analyses to quantify the influence of weather, soil conditions, and management practices on yield variation (Khaki and Wang, 2019). A transformer-based Informer model has been applied to rice yield forecasting across the Indo-Gangetic Plains by integrating time-series satellite data, environmental variables, and 2001–2016 yield records. The model outperformed multiple ML and DL baselines (R² = 0.81; RMSE = 0.41 t ha^-^¹) and achieved stable within-season accuracy (R² ≈ 0.78) as early as two months before maturity. NIRV and late-season growth stages emerged as dominant predictors, reinforcing the model’s predictive strength and interpretability (Liu et al., 2022).

Collectively, these case studies provide convergent and quantitative evidence that AI enables more accurate, earlier, and more transferable yield forecasting than traditional statistical or process-based models across satellite, UAV, and environmental data sources.

## 5 Challenges of deploying AI in real agricultural environments

Even though there have been big improvements in finding traits, detecting the environment, and predicting yields, using AI in modern farming systems is still make challenge in real conditions.

### 5.1 Data limitations and domain shift

#### 5.1.1 Data limitations

Agricultural production systems exhibit substantial spatial and temporal variability, and this complexity poses major challenges for developing reliable AI models. plant traits change across environmental conditions, reflecting the strong phenotypic plasticity of plants. Variations in day length, water supply, nutrient availability, or light intensity can lead to substantial differences in plant architecture, making the quantification of structural and developmental variation a central task in phenomics research (Poorter et al., 2019). Crop yield is influenced by interacting factors such as climate, soil conditions, fertilizer inputs, and varietal differences (Ansarifar et al., 2021). Data availability and sharing remain uneven across crops, regions, and production scales (Wu et al., 2023). HTP platforms provide valuable sensor-based phenotypic data, but most available datasets cover few sites and seasons and lack consistent formats. These limitations reduce comparability and broader use (Danilevicz et al., 2021).

#### 5.1.2 Domain shift

These data limitations exacerbate domain shift between the data-rich environments used for model training and the data-poor systems where predictions are often needed. Accurate yield estimation for major U.S. crops such as corn and soybean has become increasingly important under growing climate variability and production uncertainty. Yet these crops are grown across highly diverse agroecological zones that differ in climate patterns, soil properties and management practices. Such regional heterogeneity introduces domain, meaning that models trained in one production environment may not generalize well to others (Ma et al., 2021; Ma et al., 2023). Smallholder systems in South Asia exhibit severe spatial heterogeneity (fields are small, fragmented and managed with diverse practices), making their distribution fundamentally different from that of the large, uniform fields typically used to train remote-sensing models. Studies in the region show reduced yield-mapping accuracy under these mismatches, illustrating a clear form of spatial domain shift (Jain et al., 2016).

### 5.2 Operational, and sensor constraints

Many high-performing deep learning models require computational resources, stable data pipelines, and technical expertise that are often absent in serve farmers. Farmers and agents generally need simplified, low-maintenance decision-support tools rather than complex, opaque systems. If AI become complicated, agronomists and breeders are reluctant to trust or adopt them, and it becomes difficult to diagnose whether errors arise from sensor noise, environmental heterogeneity, or broader domain-shift effects (Gardezi et al., 2024). Sensor instability further reduces the reliability of model inputs. Imagery from UAV, proximal, and satellite platforms frequently varies in spatial resolution, spectral fidelity, temporal coverage, and calibration quality, while sensor aging and calibration drift can introduce systematic biases into repeated measurements. To mitigate these inconsistencies, combining data from multiple sensing platforms and leveraging sensor-fusion or ensemble approaches can improve robustness by averaging across individual sensor limitations (Karmakar et al., 2024; Wang et al., 2025).

Taken together, these operational, sensor, and interpretability constraints help explain why models that perform well in research environments often fail to generalize in real-world production systems.

### 5.3 Future directions

Future progress will depend on several key areas (van Dijk et al., 2021): model interpretability, which will allow predictions and explanatory outputs to align more closely with management needs (Rajpurkar et al., 2022); the incorporation of methods into multiple crop monitoring systems, enabling more consistent and timely field assessment (Jumper et al., 2021); the adoption of FAIR data principles, which will support broader multi-site datasets and improve generalizability across diverse field conditions; and (Bodnar et al., 2025) opportunities for future advancement, including streamlined architectures that can be deployed in resource-limited settings and integrative approaches that enhance operational decision support.

Looking ahead, continued improvements in data quality, model design, and agronomic integration will further enhance the practical use of AI in agriculture. Expanded multi-site datasets will support cross-regional generalization, lightweight architectures will ease deployment constraints, and advances in interpretability will allow models to provide explanations that better match real management requirements.

## 6 Conclusion

The amalgamation of AI with imaging technologies and multi-source data is revolutionizing plant phenotyping from a conventional low-throughput, labor-intensive constraint into an intelligent and data-driven high-throughput framework. Progress in various AI architectures has significantly improved the precision and flexibility of contemporary agriculture.

## 7 Discussion for perspectives

This review combines modeling methods with plant phenotyping and multi-source remote sensing to provide predictions. There are still both challenges and perspectives for future.

### 7.1 Model interpretability

Techniques such as gradient-weighted class activation mapping (Grad-CAM) (Selvaraju et al., 2017), and SHAP analysis (Desloires et al., 2024) help to clarify the impact of specific characteristics or regions on model predictions. Although the lack of consistent data, limited computing power, and poor model interpretability continue to be major obstacles to the widespread use of modeling approaches in agriculture. To get over these problems, people are now trying to lightweight analytical tools that are easier to test and use, as well as more flexible and understandable for different types of production systems, which, will meet the needs of real-world agricultural systems.

### 7.2 Multiple crop monitoring systems for remote sensing

CMS have been established worldwide to support agricultural management and food security assessment. Representative platforms include FAO’s ASIS, the European Commission’s Anomaly Hotspots of Agricultural Production (ASAP) and Monitoring Agricultural Resources (MARS), Group on Earth Observations Global Agricultural Monitoring Initiative (GEOGLAM)’s Crop Monitor, NASA Harvest’s Global Agricultural Monitoring (GLAM), China’s CropWatch, USDA VegScape, and several national systems such as FASAL in India and VEGA in Russia. These platforms share a common focus on monitoring crop growth conditions, assessing environmental drivers (Wu et al., 2023).

Other platforms provide a comprehensive and flexible foundation for agricultural and environmental observation data. Satellite platforms (MODIS, Sentinel-2, Landsat-8/9, PlanetScope, WorldView, GF series) offer broad spatial coverage and time series. UAV-based platforms can deliver centimeter-level spatial resolution and flexible flight scheduling, making fine-scale structures detection. Radar and microwave platforms (Sentinel-1 SAR, RADARSAT, ALOS PALSAR, SMAP) provide all-weather observation that are unaffected by cloud cover or illumination conditions, soil moisture mapping and crop structural analysis.

Most systems rely on time-series profiling, and spatial analysis to evaluate crop status. However, differences in classification standards continue to limit the accuracy of these systems across countries, so collaboration will promote standardized protocols and data sharing, enabling more transparent and comparable crop monitoring practices globally.

### 7.3 FAIR data principles

Data sharing and standardization is necessary to accelerate plant phenotyping and sensing. Sensing technologies and methodologies have progressed, but sensed data often differ between platforms and thus may be fragmentary and inconsistent. Shared standards like FAIR, making data comparable so that modeling traits different crop types, regions, and environmental conditions be possible.

Setting up a unified open data platform will facilitate the sharing and permanent preservation of research products. There will be more shared code in programming languages, more shared common parameters for models used in research, and a single metadata framework to express all the datasets of projects accumulated with time in different locations. So we will certainly face fewer technical barriers when working and collaborating with each other in different locations.

For future, internationally standardized agricultural data interoperable can be developed for reproducible research, use the information permanently in order to get the best value from these agricultural data sources through long-term use.

### 7.4 Opportunities for future advancement

Future research in plant phenotyping and yield prediction is expected to place increasing emphasis on integration, standardization, and interpretability, ensuring that phenotypic, environmental, and genomic data are findable, accessible, interoperable, and reusable across platforms and institutions, which will facilitate large-scale collaboration, enable more rigorous cross-study comparisons, and reduce barriers for data reuse.

Moreover, AI is entering the era of foundation models. These models are widely used, to truly understand and manage foundation models, close interdisciplinary collaboration is needed, involving not only technical aspects but also in social, ethical, and legal dimensions (Bommasani et al., 2022). In plant research, it is insufficient to concentrate alone on attaining lightweight model correctness; equal emphasis must be placed on guaranteeing the transparency and interpretability of the analytical process. It is necessary to have both of them. Finally, computer scientists, plant biologists, agronomists, and policymakers will need to work closely together to make this vision a reality. All of these approaches could help make production systems that last longer.

In summary, we propose to build an integrated architecture for global agriculture. Achieving this vision requires not only a unified, lightweight AI model but also broad collaboration. Building on open and new agricultural ecosystems with AI-based phenotyping and prediction will be certainly to revolutionize the process of generating brand new systems within the next several decades.
